# Per-pass analysis of acute ischemic stroke clots: impact of stroke etiology on extracted clot area and histological composition

**DOI:** 10.1136/neurintsurg-2020-016966

**Published:** 2020-12-09

**Authors:** Seán Fitzgerald, Rosanna Rossi, Oana Madalina Mereuta, Duaa Jabrah, Adaobi Okolo, Andrew Douglas, Sara Molina Gil, Abhay Pandit, Ray McCarthy, Michael Gilvarry, Dennis Dunker, Annika Nordanstig, Erik Ceder, Petra Redfors, Katarina Jood, Niclas Dehlfors, Georgios Magoufis, Georgios Tsivgoulis, Waleed Brinjikji, David F Kallmes, Alan O'Hare, Sarah Power, Paul Brennan, Jack Alderson, András Nagy, Ágnes Vadász, Klearchos Psychogios, Istvan Szikora, Turgut Tatlisumak, Alexandros Rentzos, John Thornton, Karen M Doyle

**Affiliations:** 1 Department of Physiology, National University of Ireland Galway, Galway, Ireland; 2 CÚRAM - SFI Centre for Research in Medical Devices, National University of Ireland Galway, Galway, Ireland; 3 Cerenovus, Galway Neuro Technology Centre, Johnson and Johnson Medical Devices, Galway, Ireland; 4 Department of Interventional and Diagnostic Neuroradiology, Sahlgrenska University Hospital, Göteborg, Sweden; 5 Department of Radiology, University of Gothenburg Institute of Clinical Sciences, Göteborg, Sweden; 6 Department of Neurology, Sahlgrenska University Hospital, Göteborg, Sweden; 7 Department of Clinical Neuroscience, University of Gothenburg Institute of Neuroscience and Physiology, Göteborg, Sweden; 8 Department of Interventional Neuroradiology, Metropolitan Hospital Athens, Piraeus, Greece; 9 Second Department of Neurology, “Attikon” Hospital, School of Medicine, National and Kapodistrian University of Athens School of Medicine, Athens, Greece; 10 Department of Radiology, Mayo Clinic, Rochester, Minnesota, USA; 11 Interventional Neuroradiology Service, Department of Radiology, Beaumont Hospital, Dublin, Ireland; 12 Department of Neurointerventions, National Institute of Clinical Neurosciences, Budapest, Hungary

**Keywords:** thrombectomy, atherosclerosis, intervention, stroke

## Abstract

**Background:**

Initial studies investigating correlations between stroke etiology and clot composition are conflicting and do not account for clot size as determined by area. Radiological studies have shown that cardioembolic strokes are associated with shorter clot lengths and lower clot burden than non-cardioembolic clots.

**Objective:**

To report the relationship between stroke etiology, extracted clot area, and histological composition at each procedural pass.

**Methods:**

As part of the multi-institutional RESTORE Registry, the Martius Scarlett Blue stained histological composition and extracted clot area of 612 per-pass clots retrieved from 441 patients during mechanical thrombectomy procedures were quantified. Correlations with clinical and procedural details were investigated.

**Results:**

Clot composition varied significantly with procedural passes; clots retrieved in earlier passes had higher red blood cell content (H4=11.644, p=0.020) and larger extracted clot area (H4=10.730, p=0.030). Later passes were associated with significantly higher fibrin (H4=12.935, p=0.012) and platelets/other (H4=15.977, p=0.003) content and smaller extracted clot area. Large artery atherosclerotic (LAA) clots were significantly larger in the extracted clot area and more red blood cell-rich than other etiologies in passes 1–3. Cardioembolic and cryptogenic clots had similar histological composition and extracted clot area across all procedural passes.

**Conclusion:**

LAA clots are larger and associated with a large red blood cell-rich extracted clot area, suggesting soft thrombus material. Cardioembolic clots are smaller in the extracted clot area, consistent in composition and area across passes, and have higher fibrin and platelets/other content than LAA clots, making them stiffer clots. The per-pass histological composition and extracted clot area of cryptogenic clots are similar to those of cardioembolic clots, suggesting similar formation mechanisms.

## Introduction

A recent study of patients with acute ischemic stroke (AIS) from the MR CLEAN clinical trial investigators demonstrated that compared with cardioembolic strokes, non-cardioembolic strokes are associated with the presence of a hyperdense artery sign, longer thrombi, and a more considerable clot burden on CT imaging, and a shift towards a more proximal thrombus location.^1^ In agreement, Dutra *et al* showed that proximal location, higher clot burden, and longer clot length are associated with worse functional outcomes and a longer endovascular procedure.^2^ These differences in clot length and burden affecting procedural and patient outcomes are probably related to the etiology of the occlusive clot. Studies investigating the histological composition of AIS clots in relation to stroke etiology have proved conflicting to date, limited by differing histological staining and analysis methods.^3–5^ In addition to variation of the histological clot composition with etiology, it has also been shown that composition varies with increasing number of procedural passes.^6^

In this large multicenter study, the per-pass histopathological composition and extracted clot area were studied in a series of patients with large vessel occlusion. The relationship between suspected stroke etiology, histological composition, and extracted clot area at each procedural pass was investigated.

## Methods

### Patient selection and clinical data

This study included patients with AIS from four hospitals collected between March 2018 and November 2019 as part of the RESTORE Registry. This study was approved by the National University of Ireland Galway research ethics committee (Study No: 16-SEPT-08) and the regional hospital ethics committees (Beaumont Hospital, Dublin; Sahlgrenska University Hospital, Gothenburg; NICN, Budapest; Metropolitan Hospital, Athens) following the ethical standards of the Declaration of Helsinki, and a waiver of informed consent was granted. The inclusion criteria were patients with a large vessel occlusion, aged ≥18 years, who had undergone mechanical thrombectomy following relevant diagnostic procedures, and with clot material successfully retrieved in at least one procedural pass.

### Clot and data collection

Endovascular treatment was performed according to the individual institution’s routine procedures. Clots were collected in a per-pass manner, meaning that where multiple procedural passes were used to treat the patients, clot material from each pass was collected separately. Data for reperfusion outcome and suspected stroke etiology were self-reported at the centers and captured on the RESTORE Registry data abstraction form. Stroke etiology was classified using the Trial of Org 10 172 in Acute Stroke Treatment (TOAST) system: large-artery atherosclerosis (LAA), cardioembolism, a stroke of other determined etiology, and cryptogenic.^7^

### Clot processing, extracted clot area analysis, and histological staining

Histological analysis of the clot samples was performed as previously recommended.^8^ Following retrieval, clot samples were immediately fixed in 10% phosphate-buffered formalin. Samples were shipped to the Department of Physiology at the National University of Ireland Galway for analysis. On arrival, each case and corresponding per-pass procedural data were logged in the RESTORE Registry. Gross photographs of each successful procedural pass were taken using a Canon EOS 1300D camera. ImageJ software (https://imagej.nih.gov/ij/) was used to analyze the area of each fragment of clot individually. First the scale was set and then the polygon tool was used to draw a region of interest around a fragment of the clot, and the area of that fragment was measured individually. The total extracted clot area for each case is defined as the sum of the clot area from all clot fragments within a case.

Per-pass clot material was then processed using a standard tissue processing protocol, embedded in paraffin wax, and cut into 3 µm sections. Two representative sections were stained with Martius Scarlett Blue (MSB) to identify the standard clot components (red blood cells, white blood cells, fibrin, platelets/other) as previously described.^9^ Orbit image analysis (www.orbit.bio) was used to quantify the MSB stained histological composition of each procedural pass.^10 11^ To indicate each component’s extent relative to the area, the histological composition for each component was multiplied by the extracted clot area for each procedural pass (area of component (mm^2^)).

### Statistical analyses

Graphpad Prism 8 was used for statistical analysis. Clinical details were reported as median (IQ1–IQ3) or number (%) of cases. Histological components were reported as mean (%) or mean area of component (mm^2^). A Shapiro-Wilk test indicated that quantitative variables did not follow a standard normal distribution, and therefore, the non-parametric Kruskal-Wallis H test was used to assess statistically significant differences among groups.

## Results

### Baseline characteristics

Four hundred and forty-one patients were included in the study; 844 procedural passes were reported (median 1 (1–21), mean 2.0 (±1.7) per case) and the clot was successfully retrieved in 612 procedural passes (72.5% of all procedural pass attempts). Median National Institutes of Health Scale score on admission was 16 (10-20). Forty-eight percent of patients were treated with intravenous recombinant tissue plasminogen activator. Eighty-eight percent of patients had an occlusion in the anterior circulation, 10% in the posterior circulation, and 2% had dual occlusions in both the anterior and posterior circulation. Successful reperfusion (Thrombolysis in Cerebral Infarction (TICI) ≥2b) was achieved in 92.3% of patients. Suspected stroke etiologies were LAA (n=115, 26.1%), cardioembolic (n=209, 47.4%), cryptogenic (n=101, 22.9%) and other suspected etiologies (n=16, 3.6%). The mean number of passes that successfully retrieved a clot were LAA (1.60), cardioembolic (1.26), cryptogenic (1.40), and other suspected etiologies (1.44). Aspiration was used as the first-line treatment approach in 55.3% of patients and stentrievers were used as first-line treatment in 44.7%. Clinical and procedural characteristics are shown in online supplemental table 1).

10.1136/neurintsurg-2020-016966.supp1 Supplementary data

### Clot histological composition and extracted clot area varies with increasing number of procedural passes

**Figure 1:**
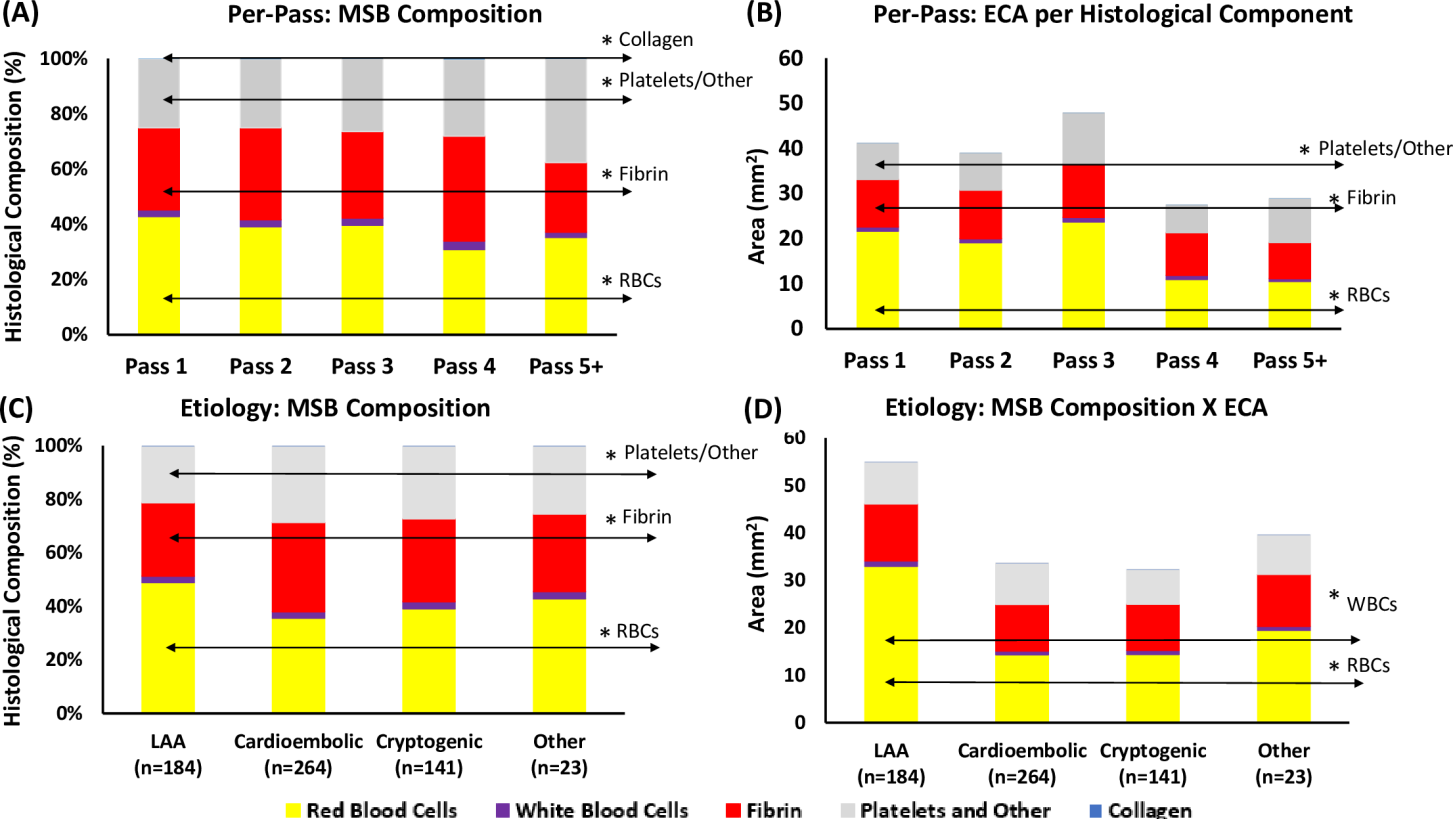
Histological clot composition and extracted clot area per histological component. (A) Mean histological clot composition (%) of each procedural pass, as determined by MSB staining represented as the percentage of the total. (B) The mean area of each histological component within a procedural pass is calculated by multiplying each component’s histological composition by the extracted clot area of the corresponding procedural pass. (C) Mean histological clot composition (%) of each suspected etiology. (D) Mean area of each histological component within each suspected etiology. ECA, extracted clot area; LAA, large artery atherosclerotic (clots); MSB, Martius Scarlett Blue; RBCs, red blood cells; WBCs, white blood cells.

Significant differences in clot composition were found with increasing number of procedural passes (figure 1A, table 1A). The proportion of red blood cells was highest in pass 1 (42.8%) and was lower in all subsequent passes (H4=11.644, p=0.020). The proportion of fibrin varied significantly across procedural passes (H4=12.935, p=0.012) and was highest in pass 4 (38.0%; figure 1A, table 1A). The proportion of platelets/other increased significantly with increasing procedural passes (H4=15.977, p=0.003), accounting for 37.4% of the composition in clots removed in passes 5+. The proportion of collagen was also significantly higher in later procedural passes (H4=10.960, p=0.027), although typically a minor component of clots. The proportion of white blood cells did not vary significantly across procedural passes (H4=1.988, p=0.738) and was also a minor component of most retrieved clots relative to red blood cells, fibrin, and platelets/other (figure 1A, table 1A).

**Figure 2:**
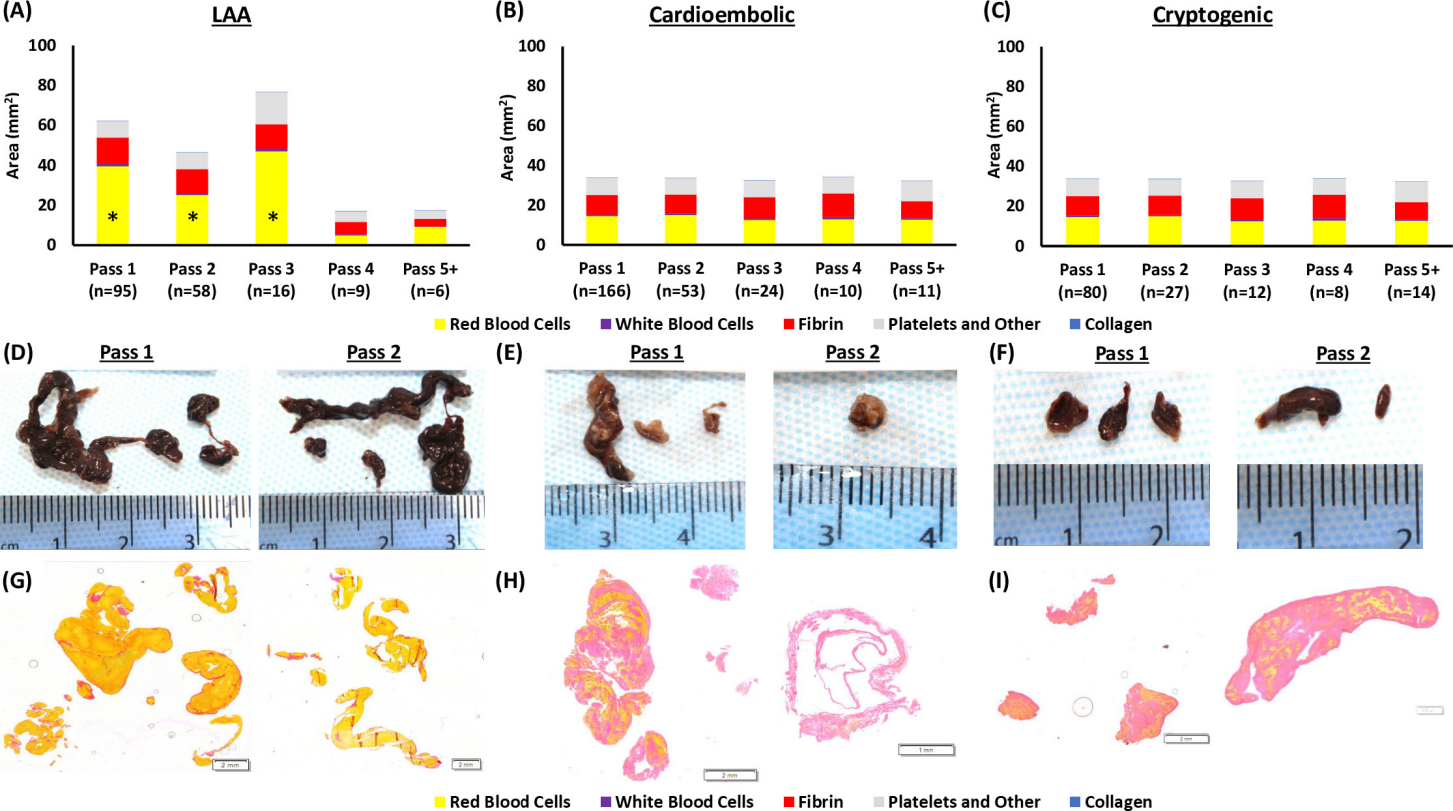
Gross photographs, MSB staining and per-pass extracted clot area of each histological component per suspected etiology. (A–C) Graphical representation of the per-pass histological composition multiplied by the extracted clot area of each suspected etiology; (A) LAA, (B) cardioembolic, and (C) cryptogenic. (D–F) Gross photographs used for extracted clot area quantification of the clots removed in passes 1 and 2 of each suspected etiology; (D) LAA, (E) cardioembolic, and (F) cryptogenic. (G–I) Corresponding MSB-stained slides used for histological quantification. *Red blood cells area in passes 1–3 of LAA clots was significantly larger than later LAA passes and all clot per pass for cardioembolic and cryptogenic etiologies. LAA, large artery atherosclerotic (clots); MSB, Martius Scarlett Blue.

**Table 1:** Histological composition per pass (A) and per-pass extracted clot area of each histological component (B)

	Total number (N)	Pass 1 (n=352)	Pass 2 (n=142)	Pass 3 (n=56)	Pass 4 (n=29)	Pass 5+ (n=33)	Test statistic (H)	DF	Significance (p<0.050*)
**(A) Per-pass MSB composition (% of total)**									
Red blood cells	612	42.78%	39.06%	39.64%	30.86%	35.20%	11.644	4	0.020*
White blood cells	612	2.43%	2.56%	2.62%	3.08%	1.92%	1.988	4	0.738
Fibrin	612	29.68%	33.34%	31.31%	38.02%	25.23%	12.935	4	0.012*
Platelets/other	612	25.00%	24.77%	26.30%	27.73%	37.43%	15.977	4	0.003*
Collagen	612	0.10%	0.27%	0.14%	0.32%	0.22%	10.960	4	0.027*
**(B) Per-pass extracted clot area per histological Component (mm** ^**2**^ **)**									
Total area	612	41.15	39.01	47.89	27.49	28.90	10.730	4	0.030*
Red blood cells	612	21.63	19.12	23.68	10.95	10.49	34.418	4	0.000*
White blood cells	612	0.93	0.85	1.05	0.88	0.61	2.218	4	0.528
Fibrin	612	10.54	10.76	11.78	9.47	8.03	13.768	4	0.003*
Platelets/other	612	8.02	8.23	11.35	6.16	9.73	19.181	4	<0.001*
Collagen	612	0.03	0.06	0.02	0.03	0.04	5.533	4	0.137

DF, degrees of freedom; MSB, Martius Scarlett Blue.

The per-pass extracted clot area per histological component showed that extracted clot area removed in earlier passes was significantly larger than in later passes (H4=10.730, p=0.030; figure 1B, table 1B). Specifically, the extracted clot area of red blood cells (H4=34.418, p<0.001), fibrin (H4=13.768, p=0.003), and platelets/other (H4=19.181, p<0.001) were all significantly different across procedural passes, with the extracted clot area of red blood cells being highest in passes 1–3, and the proportion of fibrin and platelets being highest in passes 4 and 5+ relative to the total pass area (figure 1B, table 1B). The per-pass extracted clot area of white blood cells (H4=2.218, p=0.528) and collagen (H4=5.533, p=0.137) did not vary significantly (figure 1B, table 1B).

### Clot histological composition and extracted clot area varies with suspected etiology

Significant differences in clot histology composition were found across the four etiologies: LAA, cardioembolic, cryptogenic, and other (figure 1C, table 2A). LAA clots had a significantly higher proportion of red blood cells (48.89%) than the cardioembolic (35.57%), cryptogenic (39.08%), and other etiologies (42.82%; (H3=34.418, p<0.001). The proportion of fibrin (H3=13.768, p=0.003) and platelets/other (H3=19.181, p<0.001) also varied significantly across the four etiologies with cardioembolic cases having the highest proportion of both fibrin (33.3%) and platelets/other (28.53%; figure 1C, table 2A). The proportion of white blood cells and collagen did not vary significantly across the four etiologies (H3=2.218, p=0.528, and H3=5.533, p=0.137, respectively).

**Table 2:** Histological composition per suspected etiology (A) and extracted clot area of each histological component per suspected etiology (B)

	Total number (N)	LAA	Cardioembolic	Cryptogenic	Other	Test statistic (H)	DF	Significance (p<0.050*)
**(A) MSB composition by etiology (% of total)**								
Red blood cells	612	48.89%	35.57%	39.08%	42.82%	34.418	3	<0.001*
White blood cells	612	2.42%	2.41%	2.64%	2.73%	2.218	3	0.528
Fibrin	612	27.40%	33.33%	30.96%	28.98%	13.768	3	0.003*
Platelets/other	612	21.15%	28.53%	27.13%	25.27%	19.181	3	<0.001*
Collagen	612	0.14%	0.15%	0.19%	0.21%	5.533	3	0.137
**(B) Extracted clot area X MSB composition: etiology (mm** ^**2**^ **)**								
Total area	612	54.96	33.64	32.28	39.60	13.810	3	0.003*
Red blood cells	612	32.95	14.32	14.38	19.45	27.795	3	<0.001*
White blood cells	612	1.14	0.75	0.88	0.87	7.981	3	0.046*
Fibrin	612	12.03	9.85	9.74	10.93	4.646	3	0.200
Platelets/other	612	8.79	8.69	7.26	8.32	3.054	3	0.383
Collagen	612	0.05	0.03	0.02	0.03	7.577	3	0.056

DF, degrees of freedom; LAA, large artery atherosclerotic; MSB, Martius Scarlett Blue.

The histological composition of the clots varied considerably between suspected etiologies when corrected for the area of the extracted clot material (figure 1D, table 2B). LAA clots had the largest per-pass extracted clot area at (54.96 mm^2^) and were significantly larger than cardioembolic (33.64 mm^2^), cryptogenic (32.28 mm^2^), and other (39.60 mm^2^) clots (H3=13.810, p=0.003; figure 1D, table 2B). LAA clots had the largest extracted clot area of red blood cells (32.95 mm^2^) and white blood cells (1.14 mm^2^) compared with all other etiologies (H3=27.795, p<0.001 and H3=7.981, p=0.046, respectively; figure 1D, table 2B). The overall extracted clot area of fibrin (H3=4.646, p=0.200), platelets/other (H3=3.054, p=0.383), and collagen (H3=7.577, p=0.056) were not significantly different across the four etiologies (figure 1D, table 2B).

### Variation of per-pass extracted clot area of histological components with etiology

LAA clots had a significantly larger extracted clot area of red blood cells in passes 1–3 than in passes 4 and 5+ (H4=14.527, p=0.006; figure 2A, online supplemental table 2). The extracted clot area of white blood cells (H4=7.969, p=0.093), fibrin (H4=7.783, p=0.100), platelets/other (H4=5.872, p=0.209), and collagen (H4=9.449, p=0.051) were not significantly different across procedural passes in LAA clots (figure 2A and online supplemental table 2).

LAA clots had a significantly larger extracted clot area of red blood cells in passes 1 (H3=24.290, p<0.001), 2 (H3=10.278, p=0.016), and 3 (H3=8.616, p=0.035) than all other suspected etiologies (figure 2A and online supplemental table 3). The extracted clot area of red blood cells was not significantly different between the four etiologies in passes 4 (H3=1.163, p=0.762), and 5+ (H3=1.548, p=0.671; figure 2A and online supplemental table 3).

#### Consistency of cardioembolic clots in per-pass extracted clot area and composition

The extracted clot area of white blood cells (H4=3.578, p=0.466), fibrin (H4=3.815, p=0.432), and platelets/other (H4=1.382, p=0.847) did not differ significantly across procedural passes in cardioembolic clots (figure 2B, online supplemental table 2). The per-pass extracted clot area of red blood cells was significantly different across the procedural passes in cardioembolic cases (H4=13.287, p=0.010); passes 1 and 2 had the highest extracted clot area of red blood cells. The per-pass extracted clot area of collagen was significantly different across the procedural passes in cardioembolic cases (H4=14.173, p=0.007) but the average extracted clot area of collagen was <0.1 mm^2^, and therefore it was not a major clot component.

#### Similarity of cryptogenic clots to cardioembolic clots

The per-pass extracted clot area and composition of cryptogenic clots were similar to those of cardioembolic clots (figure 2C, online supplemental table 2). The extracted clot area of red blood cells (H4=1.919, p=0.751), white blood cells (H4=2.586, p=0.629), fibrin (H4=3.006, p=0.557), and platelets/other (H4=1.674, p=0.795) did not differ significantly across procedural passes in cryptogenic clots, suggesting that the composition remains consistent throughout (figure 2C and online supplemental table 2). The per-pass extracted clot area of collagen was significantly different in cryptogenic cases (H4=20.353, p<0.001).

#### Composition and extracted clot area of other reported etiologies

The per-pass extracted clot area and composition of other known etiology clots did not vary significantly (online supplemental table 2). The extracted clot area of red blood cells (H4=3.023, p=0.554), white blood cells (H4=4.643, p=0.326), fibrin (H4=7.037, p=0.134), platelets/other (H4=5.142, p=0.273), and collagen (H4=4.794, p=0.309) did not differ significantly across procedural passes in other known etiology cases.

## Discussion

In this study, we investigated correlations between stroke etiology, histological clot composition, and clot size as determined by the area of the extracted clot. The results demonstrate that histological clot composition varies significantly with procedural passes and suspected etiology. Additionally, we show for the first time that the area of the retrieved clots varies with procedural passes and suspected etiology; LAA clots have a significantly larger extracted clot area and are richer in red blood cells in passes 1–3 than all other suspected etiologies. Fibrin and platelet/other content increases in clots removed in later procedural passes. Fibrin and platelet/other content account for a great proportion of the extracted clot area in cardioembolic and cryptogenic clots, and the composition and extracted clot area of these clots remain consistent with incremental procedural passes, suggesting a similar etiology. These findings are significant as they could have implications for the treatment of large vessel occlusion with mechanical thrombectomy, suggesting that the effectiveness of treatment could be influenced by histological composition and clot area, both of which are associated with the suspected stroke etiology.

The overall histological composition of clots retrieved from patients with AIS is heterogeneous, including red blood cell-rich, fibrin-rich, platelets/0ther-rich, and mixed clot phenotypes.^9^ Heterogeneity also occurs within a clot; a previous study of 60 cases investigating the per-pass histological composition demonstrated that the content of red blood cells was significantly higher in clots removed in passes 1 and 2 than in clots removed in subsequent procedural passes.^6^ In support of this, we found that the proportion of red blood cells is highest in clots removed in pass 1 and is lower in all subsequent procedural passes with a marked decrease at pass 4. The proportion of fibrin and platelets/other was highest in clots that took four or more passes to remove.

Increasing fibrin and platelet content leads to an increase in clot stiffness.^12^ This increased stiffness can lead to difficulties during thrombectomies, such as reduced clot ingestion by aspiration catheters and incomplete retrieval of the clot with stentrievers.^12 13^ Weafer *et al* have recently shown that the degree of clot integration into the thrombectomy device is decreased in fibrin-rich thrombi, making these thrombi more resistant to mechanical removal.^14^ This study demonstrates that the clots that are not retrieved within the first three procedural passes are fibrin and platelet-rich clots, which are probably stiffer clots and thus resistant to removal using standard first-line aspiration and stentrievers. The ultimate goal of newer generation thrombectomy devices is to achieve first-pass modified TICI 3,^15^ and novel devices are being developed specifically to treat stiff, difficult to remove fibrin- and platelet-rich clots.^16^

Initial studies investigating correlations between stroke etiology and histological composition of the retrieved clot were conflicting, often limited by small sample sizes and differing staining and analysis techniques.^4 5 17–19^ More recent studies have come to the consensus that cardioembolic thrombi have significantly fewer red blood cells and higher proportions of fibrin/platelets than non-cardioembolic thrombi.^18 20 21^ This is the largest single study on clot composition to date and in agreement, demonstrates that LAA clots have a significantly higher proportion of red blood cells overall, and in the first three passes, than all other etiologies. Fibrin, platelets/other, and collagen composition did not change significantly across the suspected etiologies in passes 1–3, suggesting that red blood cells are the component responsible for the larger extracted clot area. It has previously been suggested that non-cardioembolic strokes are associated with the presence of a hyperdense artery sign, a more considerable clot burden, a shift towards a more proximal thrombus location, and longer thrombi.^1^ This study suggests that an extremely large, red blood cell-rich clot burden is associated explicitly with an LAA source rather than a non-cardioembolic source. Patients with LAA had the highest mean number of passes that successfully retrieved a clot, suggesting that several passes are required to remove the large clot burden. LAA clots also have the largest extracted clot area of white blood cells compared with all other etiologies, which could be attributable to inflammation in the unstable atherosclerotic plaque.^22^

Patients in whom the etiology of their stroke remains undetermined following a full diagnostic investigation, accounting for up to a third of all cases of stroke,^23^ are problematic for clinicians, particularly in relation to secondary stroke prevention. Recent clinical trials are testing the efficacy of anticoagulation for the management of secondary stroke prevention, assuming that most are due to a cardiac source.^24 25^ A previous histological study demonstrated that cryptogenic strokes showed strong overlap with cardioembolic strokes but not with non-cardioembolic strokes, for both thrombus histology and interventional and clinical outcome parameters.^20^ In agreement, the findings from our study show that cardioembolic and cryptogenic clots are similar in both histological composition and extracted clot area at each procedural pass, suggesting a similar formation mechanism. Although supracardiac atherosclerosis probably accounts for a small proportion of cases of embolic stroke of undetermined source,^26^ the histological evidence suggests that the majority of such cases probably have a cardiac source.^27^

It has previously been demonstrated that diagnostic imaging is useful in identifying the composition of the occlusive clot; red blood cell-rich clots have a hyperdense artery sign on non-contrast CT, whereas platelet-rich clots appear isodense on non-contrast CT scans.^3 9 17^ Recent studies have suggested that clot-based radiomic features can also predict successful mechanical thrombectomy strategies^28^ and that patients with a hyperdense artery sign have a higher rate of first-pass effect when treated with stentrievers than with contact aspiration.^29^ With an improved understanding of the factors that influence the composition of acute ischemic stroke clots, the treatment approach may be influenced by suspected etiology in addition to diagnostic imaging. Our findings suggest that clot histological composition and clot area vary significantly with suspected etiology. Taken together, these results suggest that the suspected etiology, in conjunction with diagnostic imaging can help to predict the potential size and composition of the occlusive clot prior to treatment. Future work examining the influence of histological composition on the effectiveness of thrombectomy devices and techniques will lead to further advances. This information could inform the optimum thrombectomy strategy in each case, potentially improving the rates of first-pass effect and final revascularization outcome.

Our study has some limitations: first, only clots that were successfully retrieved from the patient were available for histopathological and extracted clot area analysis; however, successful reperfusion (TICI ≥2b) was achieved in 92.3% of patients, suggesting that most of the clot was retrieved. Second, the method described for assessing the extracted clot area is an extrapolation from a 2D image of a 3D object, which, although it will lead to some slight inaccuracies in calculating area, gives a robust relative estimate. A significant correlation between extracted clot area as measured by ImageJ and clot weight was found in a subset of samples (n=80, R^2^=0.898, p<0.05, data not shown). Third, occlusions of both the anterior and posterior circulation are included in the study, the larger vessels sizes and flow conditions of the posterior circulation might lead to more local thrombus formation, which might have unduly influenced the results of the study, although these occlusions accounted for only 10% of the cohort, with an additional 2% of cases having dual occlusions. Finally, the determination of suspected stroke etiology was self-reported at each site, and therefore there may have been some site-to-site variability in the interpretation and implementation of the TOAST criteria. We feel that the number of cases in this large cohort minimizes these effects.

## Conclusions

LAA clots are associated with a large red-blood cell clot area, particularly evident in clots collected during the first three passes of the thrombectomy procedure. Cardioembolic clots are smaller, consistently sized, and richer in fibrin and platelets than LAA clots. The area and histological composition of cryptogenic clots are similar to cardioembolic clots, suggesting a similar etiology in the majority of cases. With an improved understanding of the factors that influence the composition of acute ischemic stroke clots, the treatment approach might be influenced by suspected etiology in addition to diagnostic imaging.

## Data Availability

All data relevant to the study are included in the article or uploaded as supplementary information.
