# Supplementary material for: Per-pass analysis of acute ischemic stroke clots: impact of stroke etiology on extracted clot area and histological composition
Source: J Neurointerv Surg. 2020 Dec 9;13(12):1111–6. doi: 10.1136/neurintsurg-2020-016966 (PMC8606448; doi:10.1136/neurintsurg-2020-016966)
Supplement: Supplementary data [file neurintsurg-2020-016966supp001.pdf]

Supplementary Table 1. Clinical details of the patient cohort.

**Procedural Passes:**		
Total	844	
Clot Retrieved	612	72.52%
Mean	2.05	(±1.7)
Median	1	[1-21]
**Admission NIHSS**		
Median	16	[10-20]
**rtPA**	n	(%)
Yes	213	48.3%
No	228	51.7%
**Suspected Etiology**	n	(%)
LAA	115	26.1%
CE	209	47.4%
Unknown	101	22.9%
Other	16	3.6%
**Successfully Passes Per Etiology**	n	Average
LAA	184	1.60
CE	264	1.26
Unknown	141	1.40
Other	23	1.44
-		
**Device Used**	n	(%)
Aspiration Only	244	55.3%
Stentriever	197	44.7%

**Final eTICI:**	n	(%)
0	8	1.8%
1	4	0.9%
2a	22	5.0%
2b	115	26.1%
2c	85	19.3%
3	207	46.9%
**Territory:**	n	(%)
Anterior	390	88.4%
Posterior	41	9.3%
Both	10	2.3%
**Single Location:**	n	(%)
ACA	1	0.2%
CCA	2	0.5%
ICA	61	13.8%
M1	221	50.1%
M2	39	8.8%
M3	2	0.5%
VB	30	6.8%
PCA	8	1.8%
Multiple	77	17.50%

ACA: Anterior Cerebral Artery; CCA: Common Carotid Artery; ICA: Internal Carotid Artery; MCA: Middle Cerebral Artery, M1, M2 & M3 segments of the Middle Cerebral Artery; PCA: Posterior Cerebral Artery; OA: Ophthalmic Artery; VB: Vertebrobasilar Artery, rtPA: recombinant tissue-plasminogen activator; mTICI Score: modified Thrombolysis in Cerebral Infarction (TICI) Score. *NIHSS Data missing for 16 cases; 4 unconscious/sedated, 1 under angiography when stroke occurred, 11 data not reported on data abstraction form.

Supplementary Table 2: Per-pass ECA of each histological component for each suspected etiology.

Per Pass Extracted Clot Area X MSB Composition									
**LAA**									
	**Total Number (N)**	**Pass 1**	**Pass 2**	**Pass 3**	**Pass 4**	**Pass 5+**	**Test Statistic (H)**	**Degrees of Freedom (DF)**	**Significance (p<0.050*)**
**RBCs**	184	39.51	25.13	47.01	4.92	9.21	14.527	4	.006*
**WBCs**	184	1.32	0.96	1.45	0.50	0.22	7.969	4	.093
**Fibrin**	184	13.04	12.09	12.08	6.33	3.80	7.783	4	.100
**Platelets/Other**	184	8.43	8.39	16.15	5.22	4.15	5.872	4	.209
**Collagen**	184	0.05	0.06	0.01	0.00	0.12	9.449	4	.051
**Cardioembolic**									
	**Total Number (N)**	**Pass 1**	**Pass 2**	**Pass 3**	**Pass 4**	**Pass 5+**	**Test Statistic (H)**	**Degrees of Freedom (DF)**	**Significance (p<0.050*)**
**RBCs**	141	14.55	15.03	12.50	12.86	12.72	13.287	4	.010*
**WBCs**	141	0.76	0.69	0.72	1.20	0.73	3.578	4	.466
**Fibrin**	141	9.74	9.65	10.83	11.73	8.57	3.815	4	.432
**Platelets/Other**	141	8.76	8.28	8.54	8.27	10.23	1.382	4	.847
**Collagen**	141	0.02	0.07	0.02	0.02	0.00	14.173	4	.007*
**Cryptogenic**									
	**Total Number (N)**	**Pass 1**	**Pass 2**	**Pass 3**	**Pass 4**	**Pass 5+**	**Test Statistic (H)**	**Degrees of Freedom (DF)**	**Significance (p<0.050*)**
**RBCs**	264	16.58	12.39	9.88	16.59	8.20	1.919	4	.751
**WBCs**	264	0.89	0.88	1.05	1.10	0.53	2.586	4	.629
**Fibrin**	264	9.93	9.95	9.20	12.37	7.27	3.006	4	.557
**Platelets/Other**	264	6.25	7.80	8.14	5.96	11.98	1.674	4	.795
**Collagen**	264	0.02	0.01	0.05	0.04	0.04	20.353	4	<.001*
**Other**									
	**Total Number (N)**	**Pass 1**	**Pass 2**	**Pass 3**	**Pass 4**	**Pass 5+**	**Test Statistic (H)**	**Degrees of Freedom (DF)**	**Significance (p<0.050*)**
**RBCs**	23	10.69	31.50	38.88	6.07	18.07	3.023	4	.554
**WBCs**	23	0.59	1.02	1.44	0.13	1.75	4.643	4	.326
**Fibrin**	23	5.62	11.50	24.05	0.71	23.03	7.037	4	.134
**Platelets/Other**	23	6.07	8.13	18.71	0.65	7.91	5.142	4	.273
**Collagen**	23	0.03	0.00	0.05	0.08	0.02	4.794	4	.309

Supplementary Table 3: ECA of each histological component per suspected etiology at each procedural pass.

Pass 1								
	**Total Number (N)**	**LAA**	**Cardioembolic**	**Cryptogenic**	**Other**	**Test Statistic (H)**	**Degrees of Freedom (DF)**	**Significance (p<0.050*)**
**RBCs**	352	39.51	14.55	16.58	10.69	24.290	3	<0.001*
**WBCs**	352	1.32	0.76	0.89	0.59	5.276	3	0.153
**Fibrin**	352	13.04	9.74	9.93	5.62	4.102	3	0.251
**Platelets/Other**	352	8.43	8.76	6.25	6.07	3.647	3	0.302
**Collagen**	352	0.05	0.02	0.02	0.03	11.289	3	0.010*
**Pass 2**								
	**Total Number (N)**	**LAA**	**Cardioembolic**	**Cryptogenic**	**Other**	**Test Statistic (H)**	**Degrees of Freedom (DF)**	**Significance (p<0.050*)**
**RBCs**	142	25.13	15.03	12.39	31.50	10.278	3	0.016*
**WBCs**	142	0.96	0.69	0.88	1.02	4.049	3	0.256
**Fibrin**	142	12.09	9.65	9.95	11.50	3.938	3	0.268
**Platelets/Other**	142	8.39	8.28	7.80	8.13	0.230	3	0.973
**Collagen**	142	0.06	0.07	0.01	0.00	11.025	3	0.012*
**Pass 3**								
	**Total Number (N)**	**LAA**	**Cardioembolic**	**Cryptogenic**	**Other**	**Test Statistic (H)**	**Degrees of Freedom (DF)**	**Significance (p<0.050*)**
**RBCs**	56	47.01	12.50	9.88	38.88	8.616	3	0.035*
**WBCs**	56	1.45	0.72	1.05	1.44	4.285	3	0.232
**Fibrin**	56	12.08	10.83	9.20	24.05	2.269	3	0.518
**Platelets/Other**	56	16.15	8.54	8.14	18.71	2.537	3	0.469
**Collagen**	56	0.01	0.02	0.05	0.05	5.851	3	0.119
**Pass 4**								
	**Total Number (N)**	**LAA**	**Cardioembolic**	**Cryptogenic**	**Other**	**Test Statistic (H)**	**Degrees of Freedom (DF)**	**Significance (p<0.050*)**
**RBCs**	29	4.92	12.86	16.59	6.07	1.163	3	0.762
**WBCs**	29	0.50	1.20	1.10	0.13	3.460	3	0.326
**Fibrin**	29	6.33	11.73	12.37	0.71	8.091	3	0.044*
**Platelets/Other**	29	5.22	8.27	5.96	0.65	4.379	3	0.223
**Collagen**	29	0.00	0.02	0.04	0.08	8.209	3	0.042*
**Pass 5**								
	**Total Number (N)**	**LAA**	**Cardioembolic**	**Cryptogenic**	**Other**	**Test Statistic (H)**	**Degrees of Freedom (DF)**	**Significance (p<0.050*)**
**RBCs**	33	9.21	12.72	8.20	18.07	1.548	3	0.671
**WBCs**	33	0.22	0.73	0.53	1.75	4.049	3	0.256
**Fibrin**	33	3.80	8.57	7.27	23.03	2.271	3	0.518
**Platelets/Other**	33	4.15	10.23	11.98	7.91	3.130	3	0.372
**Collagen**	33	0.12	0.00	0.04	0.02	6.864	3	0.076
